# An Ontology-Based Chatbot to Enhance Experiential Learning in a Cultural Heritage Scenario

**DOI:** 10.3389/frai.2022.808281

**Published:** 2022-04-25

**Authors:** Mario Casillo, Massimo De Santo, Rosalba Mosca, Domenico Santaniello

**Affiliations:** Information Communication Technologies (ICT) Center for Cultural Heritage, Department of Industrial Engineering (DIIN), University of Salerno, Fisciano, Italy

**Keywords:** ontology, chatbots, cultural heritage, experiential learning, knowledge management

## Abstract

Italy is rich in cultural attractions, many known worldwide, others more hidden and unrecognized. Cultural attractions include tangible cultural assets (works of art, archaeological excavations, and churches) and intangible ones (music, poetry, and art). Today, given the pervasive diffusion of “smart” devices, the intelligent use of modern technologies could play a crucial role in changing the habit of consulting and visiting cultural heritage mainly with traditional methodologies, making little or no use of the advantages coming from the more and more availability of digitalized resources. A realm of particular interest is “experiential learning” when applied to cultural heritage, where tourists more and more ask to be helped in discovering the richness of sites they explore. In this article, we will present an innovative chatbot-based system, called HeriBot, that supports experiential tourism. Our system has been developed and experimented with a research effort for applying ICT technologies to enhance the knowledge, valorization, and sustainable fruition of the Cultural Heritage related to the Archaeological Urban Park of Naples (PAUN—Parco Archeologico Urbano di Napoli). Our article starts exploiting the ontological approach based on a purpose ontology describing the Park Heritage. Using such an ontology, we designed a chatbot that can identify the specific characteristics and motivations of the tourist, defining language, tone, and visitable scenarios and, through the ontology, allows the visit to be transformed into a personalized educational opportunity. The system has been validated in terms of dialogue effectiveness and training efficiency by a panel of experts, and we present and discuss obtained results.

## Introduction

With as many as 58 registered sites, Italy is among the top countries in the world for the number of heritages protected by UNESCO as reported in the World Heritage List.^1^ While Italy is highly visited for its cultural heritage and tourist sites, several potentials of these assets remain to be explored, and many places remain unknown due to lack of information and investments.

Even when this lack is fulfilled, some places might remain hidden due to the incapacity to convey the proper indications to tourists and help them choose destinations that, while appearing as unimportant, are treasures of beauty and culture. More and more, tourists are flooded with a large amount of unrequested information, mainly focused on some very famous “touristic attractors” and unaware of the vast heritage belonging to “minor sites” (Mendoni, 2016). Nowadays, given the pervasive diffusion of “smart” devices, the intelligent use of modern technologies could play a crucial role in modifying the above-presented scenario.

Chatbots represent a leading example (Lombardi et al., 2019). A Chatbot is a system capable of simulating and processing human conversations. Such systems allow users to interact with digital devices as if they are communicating with a person, providing the correct information at the right time. In the touristic realm, chatbots are increasingly popular for their active support. While they can provide travelers with information and suggestions on tourist and leisure spots, when linked with a suitably designed knowledge base, they make it easy to build personalized itineraries based on individual preferences and profiles.

It is also worth noticing that in this way, tourism can also be thought of as a form of learning, allowing people to learn something new through their experiences, a model called Experiential Learning. Turning a tourist visit into an educational opportunity using a chatbot can be a new form of Experiential Tourism (Ruhanen, 2005; Arcodia et al., 2021).

This work also stems from previous experiments aimed at generating personalized tourist routes, starting from data belonging to the cultural heritage of the Campania region.

In Casillo et al. (2020), the authors proposed a recommender system capable of developing adaptive tourist routes. The proposed system suggests points of interest and related services based on the tourist profile and contextual aspects of the visiting area. In this previous work, the user interaction with the system occurs through a chatbot developed to support the user in constructing a personalized tourist route related to some of the most important cultural sites in Campania. While experimenting with the quoted chatbot, the authors realized some limitations of the approach: first of all, the use of context is of limited value when operating in a homogeneous area; furthermore, profiling users could be difficult when such profiles have to been built with the explicit intervention of the user themselves. And finally, a more appropriate formalization of the knowledge about the cultural sites and the links, sometimes hidden, among concepts, stories, manufacts, etc. belonging to the cultural site could be the key to give the chatbot the capacity to conduct a more flexible and performative talk with the human user.

Starting from these considerations, in the current work we experimented the use of an ontology as a tool for accurately modeling the knowledge about the assets and the context of the cultural site under visit, also trying to design a chatbot that could respond effectively even without previously acquired user profiling. The chatbot responds efficiently to users who have never been profiled before, since it is able to categorize users (naive or expert) according to the questions they ask.

The work aims to present the results of a study conducted by implementing a chatbot whose responses are fed by a knowledge base related to the Archaeological Urban Park of Naples (PAUN).^2^ This knowledge base was built from the digitization of more than 10,000 cataloging cards derived from the archaeological excavations carried out in Piazza del Municipio in Naples.

Our article 's first original contribution consists of the description of the methodology adopted for designing a specific ontology (a so-called “purpose ontology”) starting from the availability of a more general ontology related to Italian cultural heritage. If the knowledge base has the form of an Ontology (Gruber, 1995), it is also possible to exploit hidden links among entities and help people discover unexpected relations among their preferences and destinations. Connecting a chatbot to a purpose Ontology allows the system to react by building question-answer correspondences that exploit the previously described hidden links and relations. We highlight the advantages of adopting an ontology more focused on describing the vast knowledge available on the specific heritage of the Urban Archaeological Park of Naples. In the design of the purpose ontology, it is also showed that it is possible to define different semantic levels of information, preparing the knowledge base to be consulted even from assumptions about the preferences of end-users.

The second innovative contribution of the work consists in showing how it is possible to build a well-suited correspondence between the questions of the end-users and the answers of the chatbot using a simple approach based on the use of neural networks. The chosen neural network architecture and the general architecture of our system are presented and discussed. An experiment conducted with two groups of users chosen for their different characteristics is offered to validate the hypotheses formulated in the article.

The article is organized as follows.

We start presenting a literature review about using chatbots to interact with human users and designing ontologies to capture the knowledge possessed by the Chatbot. Then, we present how using such an ontology to give life to a chatbot that can identify the specific characteristics and motivations of the tourist, defining language, tone, and visitable scenarios and, through the ontology, allows the visit to be transformed into a personalized educational opportunity. Successively, aiming to describe the exploitation of the ontological approach we adopted, we show the design of a purpose ontology describing the PAUN Heritage. Finally, we present how the system has been validated in terms of dialogue effectiveness and training efficiency by a panel of experts from the PAUN project and discuss obtained results and further studies.

## Related Works

### Chatbots

Borrowing their name from the highly successful social network “chats,” *chatbots* can be defined as agents or systems mimicking humans' dialogues so that a human user can interact with machines having the feeling to talk (or write) with another human.

The first practical application of a chatbot was with the ELIZA program (1966), created by Joseph Weizenbaum's (Fryera et al., 2019). This program aimed to reconstruct a dialogue/interaction that a psychoanalyst could have with his patient.

When working in the chatbots' realm, a crucial issue is to provide the user with the idea that the interaction is performed by a human being and not a technological system. Users, in fact, tend to pay attention to a person rather than software for reasons of habit and reliability. This issue is especially true if the intent of the chatbot designer is to provide the user with the experience to be guided along a learning path of some sort, resembling the involvement a human skilled guide can guarantee. To make the conversation through chatbots more and more fluid and to ensure that the answers to the questions posed to the system are more and more relevant, several studies and experiments have been done over the years.

In the literature there are several models to classify the functioning of chatbots (see for example, Fryera et al., 2019; Agarwal and Wadhwa, 2020; Suhaili et al., 2021). In the following, we will focus on the classification of methods for generating the answers proposed in (Agarwal and Wadhwa, 2020), where chatbots can be rule-based or neural networks-based.

Chatbots adopting a rule-based approach perform a keyword analysis of the question to generate the answer using pre-filled response templates. In this way, a database of questions and answers is created, which the system draws upon in its operation. The main techniques used for the Rule-Based Approach model are Pattern Matching (Suhaili et al., 2021), Parsing (Adamopoulou and Moussiades, 2020), Markov Chain Models (Lokman and Jasni, 2010), Semantic Nets (Shum et al., 2018), AIML (Adikari et al., 2019), Structured Query Language (SQL), and Relational Database Language Tricks (Augello et al., 2009; Petrovski et al., 2018).

As often happens in the application of machine learning techniques, these systems make use of a continuous process of enrichment of the database of questions and answers, and their behavior is very much linked to the intervention of the so-called “boot master,” a human supervisor who checks the conversations and updates / modifies the database to ensure that the answers are as relevant as possible to the questions the user asks (Thomas, 2016).

More recent developments have led to the birth of models for chatbots' design based on the use of neural networks using deep learning technology. The neural network is trained on suitable datasets to generate relevant and grammatically correct responses. The neural network-based approach can be further classified as retrieval-based and generative. In retrieval mode, the chatbot retrieves the replies from a set of previously defined answers. In the generative mode, the responses can also be directly generated by the system. The interested reader can find interesting examples of neural network based chatbots in Al-Zubaide and Issa (2011); Wu and Yan (2019); Karna et al. (2020); Sperlì (2020); Dhyani and Kumar (2021).

### Semantic Web and Ontology

To have a high quality chatbot, a well-designed and in-depth knowledge base needs because the system must have as detailed information as possible. Another problem arises when the knowledge base must be populated with the origin of the data. With the growth of the Internet, one of the most popular means of acquiring news and information is the Internet. However, the data found on the network are present in forms and languages that are many times different from each other, and, in order to standardize them, special dedicated programs are required.

One interesting and powerful approach to manage the knowledge built in the chatbot is coming from Semantic Web studies. It was Berners Lee in 2001 who introduced the word “Semantic Web,” a technology that aimed to overcome the limitations set out above. In particular, the information search, cataloging and extraction operations can be carried out automatically by computers through the presence in the text of hyperlinks and key terms (Berners-Lee et al., 2001).

Bakouan et al. (2018) proposed a schematic view of the semantic web distinguishing it in three levels:

- A Naming/addressing Level where URI (Universal Resource Identifiers) are used;
- A Syntactic Level, where XML and Name spaces are introduced;
- A Semantic Level where concepts and links among them are defined in a common standardized way so to enable interoperability and automatic reasoning. At this level, tools like RDF and Ontology are introduced.

The semantic level is of particular importance for the correct functioning of chatbots, and ontology is the key tool for better populating the knowledge base.

Under the concept of ontology we conceive a form of knowledge representation that consists of two elements necessary to structure the so-called “semantic network:” concepts and the relationships between them (Noy, 2004).

The three main components of an ontology are: Terms, logical axioms, and the ontology language best suited to the purpose for which the ontology is being developed.

An axiom can be defined as the specification of a class of objects and the relationships that exist between them. Axioms are fundamental elements, because they describe what is true in a domain, they enable computational inferences, they can reduce repetition in knowledge representation, they facilitate ontology development and updating.

In a nutshell, ontologies are frameworks for representing shareable and reusable knowledge in a domain. Their ability to describe relationships and their high interconnectedness make them the foundation for modeling high-quality, connected, and consistent data.

Among the various types of ontologies, a purpose ontology provides domain-specific terms related to set purposes. This allows a specific domain to be used in a way that best suits the intended purpose. In our case study, data from the Naples Urban Archaeological Park was modeled in a purpose ontology to make it useful for both general and expert users.

In the literature, several articles investigate the use of ontologies for cultural heritage (Hernández et al., 2008; Pattuelli, 2011; Rossana Damiano et al., 2014; Ellefi et al., 2018), while to the best of our knowledge chatbots for cultural heritage have only recently begun to be explored. One interesting example can be found in Sperlì (2021).

## Ontology Guided Chatbot

As discussed above, our approach exploits an ontology guiding the flow of the conversation between the human user and the chatbot.

Adopting a purpose ontology is the right choice to achieve our desired goals. In Section Designing PAUN Ontology, we discuss in depth this assumption.

The ontology will be designed to map the whole knowledge domain of interest accurately. Starting from this knowledge, the chatbot will understand and react to the user's interests through a database of questions and answers extracted from the ontology by using a neural network.

A conversation can be seen as a sequence of questions/answers from the user to the chatbot and vice-versa. In this way, it is possible to map the single interaction to establish the topic and its attributes, so modeling a conversational workflow and formalizing the problem analytically.

During dialogue, the Chatbot consults the ontology through queries modeled on different levels of detail. Within the ontology, the concepts are grouped with labels that allow different levels of knowledge to be differentiated. A more general textual knowledge is then associated with the domain of interest. The main advantage of ontology is that it makes it possible to explore the conversational paths more flexibly.

### Architecture of Our System

Let us start describing the architecture of the proposed Chatbot based system, called HeriBot (Cultural **Heri**tage Chat**Bot**). This architecture allows to interact with users in a natural way and provides personalized information. Through textual interaction, Heribot will guide the user with three main objectives:

- Identify and provide for the user requests according to the different belonging categories.
- Guide the user in the selection of the various activities based on the logic of satisfaction.
- Interact with the user concerning specific interests expressed during the conversation.

A crucial issue is to provide the user with the idea that the interaction is performed by a human being and not a technological system. Users, in fact, tend to pay attention to a person rather than software for reasons of habit and reliability. Another technological challenge is to understand the intent within a sentence introduced by the user, which represents the authentic inquiry submitted to the Chatbot.

**Figure 1:**
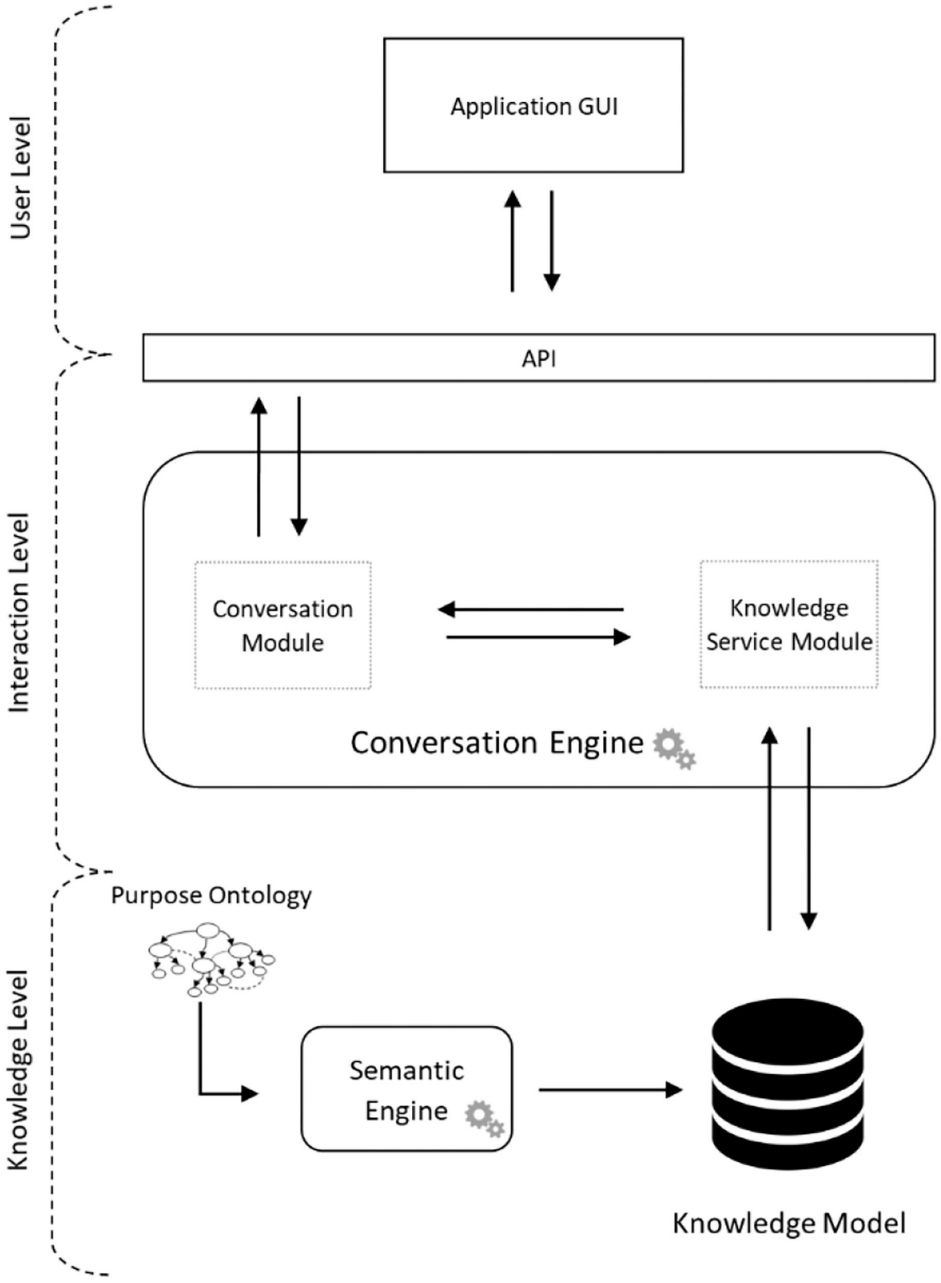
System architecture.

The proposed architecture is described in Figure 1, in which the three main layers are represented by:

- User level: the level at which users can make requests and receive responses from the HeriBot.
- Interaction level: the level where the interactions of the Chatbot with users are generated.
- Knowledge level: the level where all the information that the system can provide are located and managed.

At the User level, the interaction with HeriBot is performed through the Graphical User Interface (GUI) and the Application Programming Interface (API). The API is responsible for user communication with the system, handling requests, and constantly communicating with the Conversation Module located inside the Conversation Engine located at the Interaction Level.

The Conversation Engine represents the HeriBot core implementing dialogues through the help of two sub-modules: Conversation Module and Knowledge Service Module. The Conversation Module dialogues with the user through conversation templates. Based on the information contained in the questions proposed by the user, HeriBot can access the conversational templates that lead to the satisfaction of the user's intent. To this aim, the Conversation Module interacts with the Knowledge Service Module receiving the conversation template tailored to the user's need. The Knowledge Service Module queries the Knowledge Model obtaining a conversation template.

The Knowledge Level contains the knowledge database, characterized by the processed knowledge extracted from the Purpose Ontology. The Knowledge Model contains all possible dialogue patterns arising from the purpose ontology. The Knowledge Service Module uses it to advance the dialogue by making it natural and reliable.

**Figure 2:**
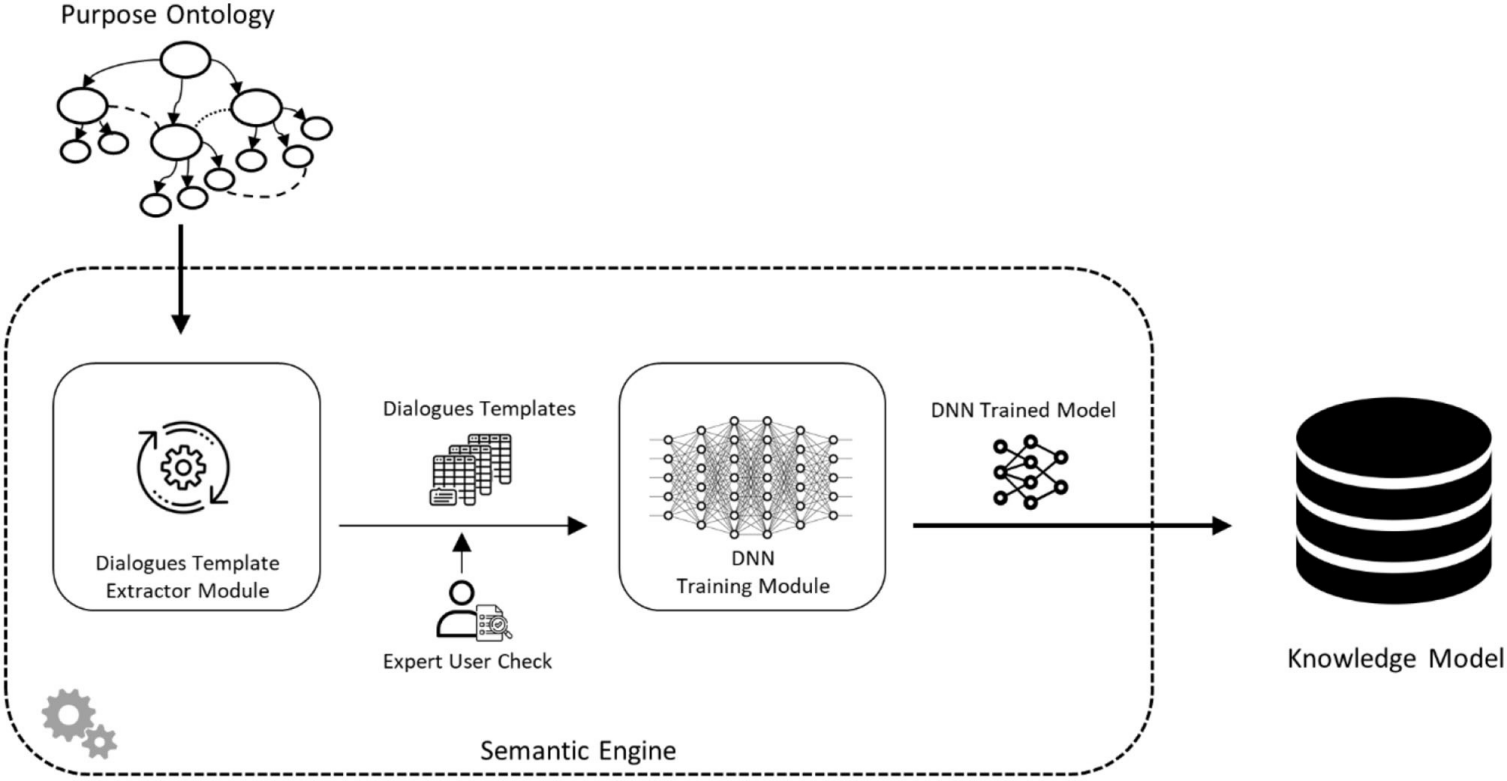
System knowledge model workflow.

Figure 2 shows the workflow for the construction of the Knowledge Model that is exploited by the chatbot. In the figure, the process of knowledge extraction from the ontology is represented. The Purpose Ontology, which describes the reference domain, is exploited by automatically extracting all possible dialogue templates concerning user intent, the complexity of the conversation, and the topic. After the extraction, the dialogue templates are validated by the ontology designers. The dialogue templates are organized by Natural Language Processing techniques to train the proposed system through a Deep Neural Network (Schmidhuber, 2015).

Through this approach, HeriBot can understand the user's needs and requests, understand the relevant topic, and change the level of interaction based on the user's profile.

## Designing PAUN Ontology

In the present Section, we discuss in depth the design of our purpose ontology. We can point out the following issues:

1. Chatbots are widely recognized as a good tool to naturally interact with humans
2. Ontologies promise to be an efficient mean to empower chatbots knowledge and ability to dynamically adapt their answers to requests of the human user
3. Suitably designing the PAUN Ontology is the main challenge to face for obtaining the previous goals.

The domain ontology of PAUN (Urban Archaeological Park of Naples) was modeled with the Protégé ontology editor.^3^

### Ontology Design Methodology

The aim of our ontology is to help experts and tourists in the fruition of the Cultural Heritage of the Archeological Urban Park of Naples (PAUN). We started from the data contained on the DatabencArt Platform (DAP). DAP is a Cultural Heritage oriented software platform developed by the High Technology District for Cultural Heritage^4^ and used in various research projects (Cornevilli et al., 2020). DatabencArt contains about 20,000 records related to the Cultural Heritage of the Campania region. More than 10,000 regard the area called “Piazza Municipio” where the Archeological Urban Park is located. The interested reader can access the PAUN Project website (see text footnote ^2^) where she can find a detailed description of the Park.

We started modeling the ontology following the methodology proposed in Noy and McGuinness (2001).

In our specific case, the steps were as follows:

(1) Determination of the domain and purpose of the ontology
(2) Study of existing ontologies in the Cultural Heritage domain
(3) Investigating the re-use of existing ontologies
(4) Enumeration of important terms in the ontology
(5) Definition of classes and class hierarchy
(6) Definition of the properties of slots of the classes
(7) Creation of instances.

The construction process of our ontology is iterative and incremental. It means that it is possible to revise and refine the model in addition to our case study for data collection. The obtained model is consistent and well-adapted to the variety of data input. A further step follows of building inference rules to discover more relationships systematically.

Following step (2) of the adopted design methodology, we investigated the realm of Cultural Heritage oriented ontologies (Kakali et al., 2007; Doerr, 2009; Nafis et al., 2019).

Among them, the ArCo initiative is worth mentioning (Carriero et al., 2019). In short, ArCo (“**Ar**chitettura della **CO**noscenza,” Architecture of Knowledge in Italian) is the Knowledge Graph of the Italian cultural heritage and includes a network of seven vocabularies that describe the domain of cultural heritage and the data extracted from the General Catalog of Cultural Heritage of ICCD-MiBAC.^5^ The ontological network of ArCo is built by seven ontological modules connected by import axioms. Two modules—called Arco and Core—include high-level concepts and generic relationships between modules, respectively. The remaining five modules focus on cultural assets and their characteristics.

The first phase of our work to model the ontology involved a careful exploration of the ontology modules of ArCo. Then, an original mapping was created between the fields of the records present on the DatabencArt platform and the fields of the ontology modules of ArCo. This allowed us to build a first rudimentary ontology, which was then expanded and modeled on the specific knowledge of the PAUN Park of Naples.

### Designing Our Purpose Ontology

Our purpose ontology is restricted to concepts, relationships, and knowledge extracted from studies on the Archaeological Urban Park of Naples. Our study focuses on the use of ontology to represent knowledge and answer the following questions:

- How can this sectoral knowledge be made available?
- What kind of information is suitable for a general user?
- What types of information are suitable for a sector expert?
- What are the basic requirements for consulting this information?
- What types of curiosity can a tourist have?
- What links between the data might interest an archaeologist/art historian/geologist?

Two main resources, which help us to enumerate important terms in the ontology, are:

(1) the data repository contained in DAP
(2) The mapping between the classes already used in ArCo

From the DAP database, we have collected some terms, which correspond to those provided by the records. Among the main classes we find movable property, immovable property, photographic attachments, technical attachments. All the classes and their relations in our ontology aim to represent the data contained in DAP, collected by the experts, and made available by the Superintendence for Architectural Heritage and Landscape of the City of Naples.

Let us now explain how the data stored in the DatabencArt platform has been incorporated into our ontology.

To model our ontology, we started with a careful exploration of the ontological modules of ArCo. We then mapped all the fields of the records contained in DatabencArt with classes or properties present in ArCO. Table 1 shows the quoted mapping. In the table, the sign “√” indicates the correspondence of the DatabencArt field to a concept of the ArCo ontology, while “X” indicates the absence of correspondence.

**Table 1:** Correspondences between the modules of ArCo ontology and records of DatabencArt.

**Records**	**Ontological fields**								
**Type of**	**Movable/**	**Tecnichal**	**Cultural**	**State of**	**Acces**	**Property compiled**	**The class specifications**	**Catalog**	**Legal**
**records**	**imovable**	**characteristic**	**domain**	**conservation**	**profile**	**at the**	**of the cultural**	**sheets**	**status**
**DatabencArt**	**property**	**(MTC)**	**(ATB)**	**(STC)**	**(ADS)**	**time (CMP)**	**property (OGT)**	**(TSK)**	**(TU-COND)**
A	✓	✕	✓	✕	✓	✓	✓	✓	✓
CA	✓	✕	✕	✕	✓	✓	✓	✓	✓
MA	✓	✕	✕	✕	✕	✓	✓	✓	✓
MI	✓	✓	✓	✓	✕	✓	✓	✓	✓
OA	✓	✓	✓	✓	✓	✓	✓	✓	✓
RA	✓	✓	✓	✓	✓	✓	✓	✓	✓
SAS	✓	✕	✕	✕	✓	✓	✓	✓	✕
TMA	✓	✕	✕	✕	✕	✓	✓	✓	✕
US	✓	✕	✕	✓	✓	✓	✓	✓	✕
USM	✓	✕	✕	✓	✓	✓	✓	✓	✕

As you can see from the Table 1, DatabecArt contains very detailed knowledge that is lost in ArCo. ArCo designers have modeled very general categories and, consequently, some of the fields in the DatabencArt records cannot be easily mapped.

Let us further explore this issue. Table 2 highlights the mapping on ArCo of a single RA record (Reperto Archeologico—Archaeological Finds) contained in DatabencArt. The table shows that some of the fields of the quoted record are not present in ArCo. To facilitate consultation, we have highlighted the missing fields in yellow. It is evident that the mapping on ArCo helped model some concepts and properties but was insufficient to represent our knowledge domain fully.

**Table 2:** Correspondences between the fields of the DatabencArt RA type record and the ArCo ontology nodes and properties.

**DatabencArt**	**ArCo**
Tipso oggetto/bene mobile	Arco/Cultural Entity/Bene culturale/Bene Materiale/Bene mobile
MT-Dati Tecnici	Arco/Entity/Characteristic/Caratteristica tecnica (MT/MTC)
MTC-Materia e tecnica	
AD-Accesso Dati	Denotative-description/Entity/Characteristic/Profilo di accesso (AD/ADS)
ADS-Specifiche di accesso	
CO-Conservazione	Denotative-description/Entity/Object/Concetto/Tipo/Tipo di conservazione (CO/STC/STCC)
STC	
STCC	
DT-Cronologia	Arco/Object properties/ha cronologia (DT)
DTZ	
DTZG	
CM-Compilazione	Arco/Object properties/compilato al tempo (CM/CMP/CMPD)
CMPD	
CMPN	
AU-Def Culturale	Arco/Topic/Ambito culturale (AU/ATB)
ATB	
ATBM	
ATBD	
LC-Localizzaz-geo	Arco/Object properties/ha localizzazione tipizzata nel tempo (LC)
LDC	
LDCN	
LDCU	
OG-Oggetto	Arco/Entity/Description/Specifiche del bene culturale (OG/OGT/OGTT)
OGT	
OGTN	
OGTD	

*For the sake of clarity, we report the English translation of the names of the missing fields in ArCO. The quoted fields are highlighted in yellow. DTZG “Chronology of the asset;” CMPN “Compilation of the form;” ATBM, ATBD “Cultural Area;” LDC, LDCN, LDCU “Geographic-administrative location;” OGTN, OGTD “Definition of the object”*.

Therefore, we decided to model a purpose ontology, where we added all the specific subfields present in DatabencArt.

To achieve this goal, we have studied each record contained in DatabencArt Platform relating to the Urban Archaeological Park of Naples.

PAUN records are divided into two distinct domains named respectively “Castel Nuovo” and “Piazza Municipio.” This division was chosen by domain experts for more efficient consultation. In Tables 3, 4, we show these two domains reporting all types of records and their quantity.

**Table 3:** Representation of the ICCD cards belonging to the “Scavo Piazza Municipio” domain.

**ICCD card typologies**	Number
MA (Archaeological Monument)	379
US (Stratigraphic Unit)	5.874
USM (Masonry Stratigraphic Unit)	2.471
USR (Stratigraphic Unit of Reference)	711
TMA (Materials Table)	170
RA (Archaeological Find)	124
**ICCD annexes**	Number
Photographic and technical attachments	37.262

*The table puts into evidence that the records are linked to photographic and technical attachments*.

**Table 4:** Representation of the ICCD cards belonging to the “Castel Nuovo” domain.

**ICCD typologies**	Number
A (Architecture)	17
US (Stratigraphic Unit)	746
USM (Masonry Stratigraphic Unit)	325
USR (Stratigraphic Unit of Reference)	4
SAS (Stratigraphic Essay)	3
RA (Archaeological Find)	9
OA (Artwork)	12
**ICCD annexes**	Number
Photographic and technical attachments	73

*The table puts into evidence that the records are linked to photographic and technical attachments*.

The above-mentioned distinction in two domains has been maintained within our purpose ontology to facilitate a greater expressive capacity.

**Figure 3:**
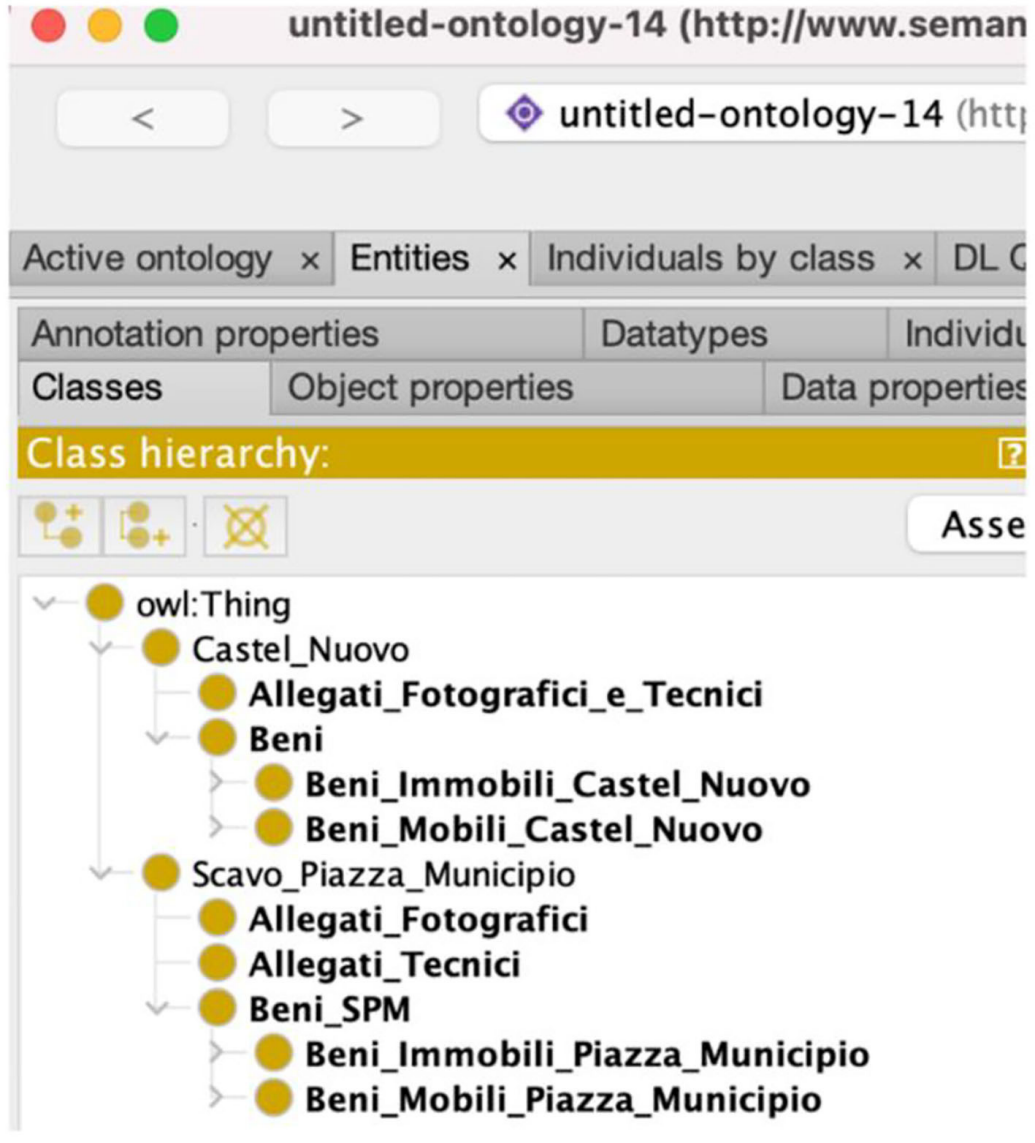
A detail of the PAUN ontology showing the superclasses “Castel Nuovo” and “Scavo Piazza Municipio” which reflect the two domains corresponding to the main areas of the Urban Archaeological Park of Naples.

Figure 3 shows a detailed view of our ontology where we can see the correspondence between cataloging in DatabencArt and modeling in ontology. In particular, the first superclasses are an expression of the two domains corresponding to the above-mentioned main areas of the Park.

**Figure 4:**
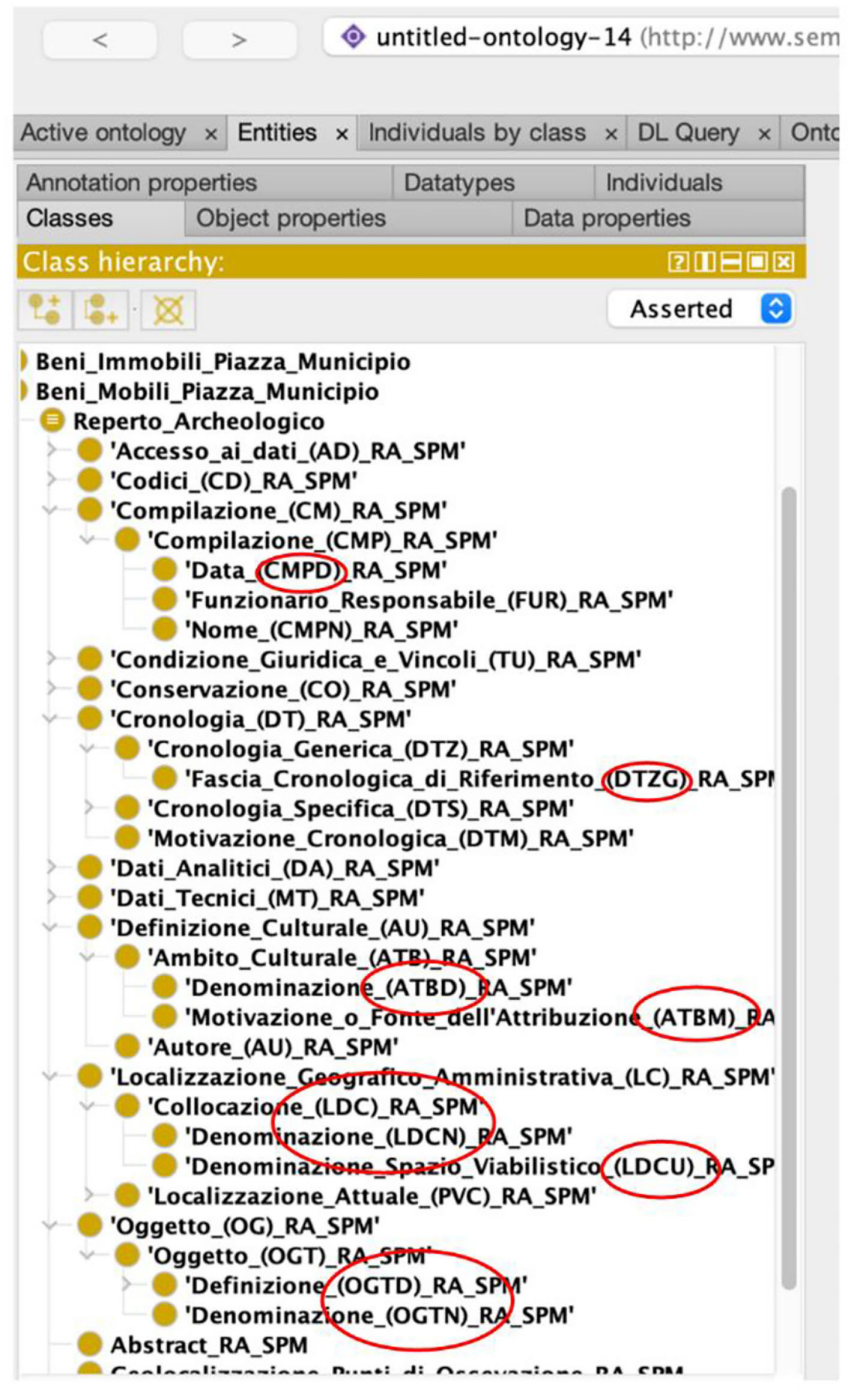
A detail of the PAUN ontology mapping the RA “Archaeological Find” record. We circled in red the nodes of the ontology not found in ArCo.

Figure 4 shows a detailed view of PAUN ontology modeling the RA “Archaeological Find” record. In the figure, all record subfields missing in ArCo (as previously underlined in Table 2) are introduced.

Let us examine some examples showing how the detailed description of the records contained in DatabencArt helped construct of the inference rules in our purpose ontology.

The first case regards a naive user who knows nothing about the Urban Archaeological Park of Naples. As a starting point, she will want to know which assets are present to go and visit them.

To get this inference, we have:

- modeled the property “belongs to” that connects the single property to its domain of belonging (Castel Nuovo or Scavo Piazza Municipio).
- applied a restriction to ensure that each described asset belongs only to the superclass “Castel Nuovo” or only to the superclass “Scavo piazza Municipio.”

**Figure 5:**
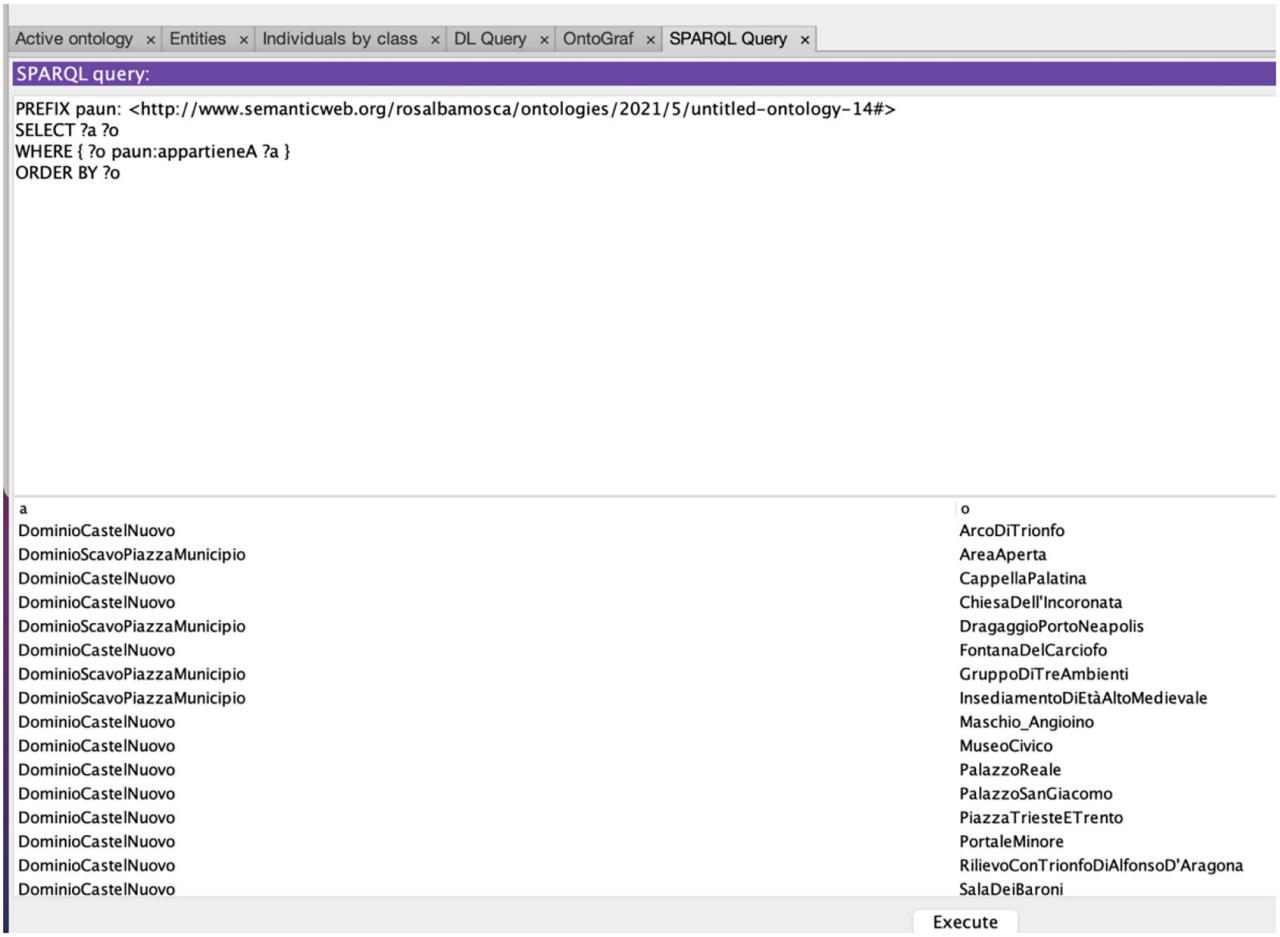
Query on PAUN Ontology showing each asset in the domain it is placed in.

The result of this inference is shown by the query reported in Figure 5.

The answer contains: (1) all the assets present in PAUN, (2) the assets divided into the two domains (Castel Nuovo and Scavo Piazza Municipio), (3) the assets in alphabetical order to facilitate their consultation.

This way of correlating information within the ontology allows the user to obtain targeted information and design interesting tour itineraries within the PAUN. In fact, with the growth of the user's competence and curiosity, the particularity and complexity of the proposed routes increases.

As a second case, we assume that an expert user wants to discover uncommon paths or relate findings coming from different eras.

To illustrate this scenario, let us take a record of the “stratigraphic unit” (US) type.

In the case of the records of the “Stratigraphic unit” type, a dialogue with the designers of DAP highlighted the need to:

- relate cards belonging to different historical periods,
- compare different materials,
- examine the different conservation states.

Consequently, we modeled many properties that meet these needs in PAUN Ontology. For example, we introduced the property “has state of conservation.” This property links each stratigraphic unit to a particular state of conservation: not available, bad, good, and excellent. In this way, this property makes it possible to make a particularly useful inference for experts users (e.g., archaeologists) who can relate stratigraphic units of different states of conservation in which they are found.

**Figure 6:**
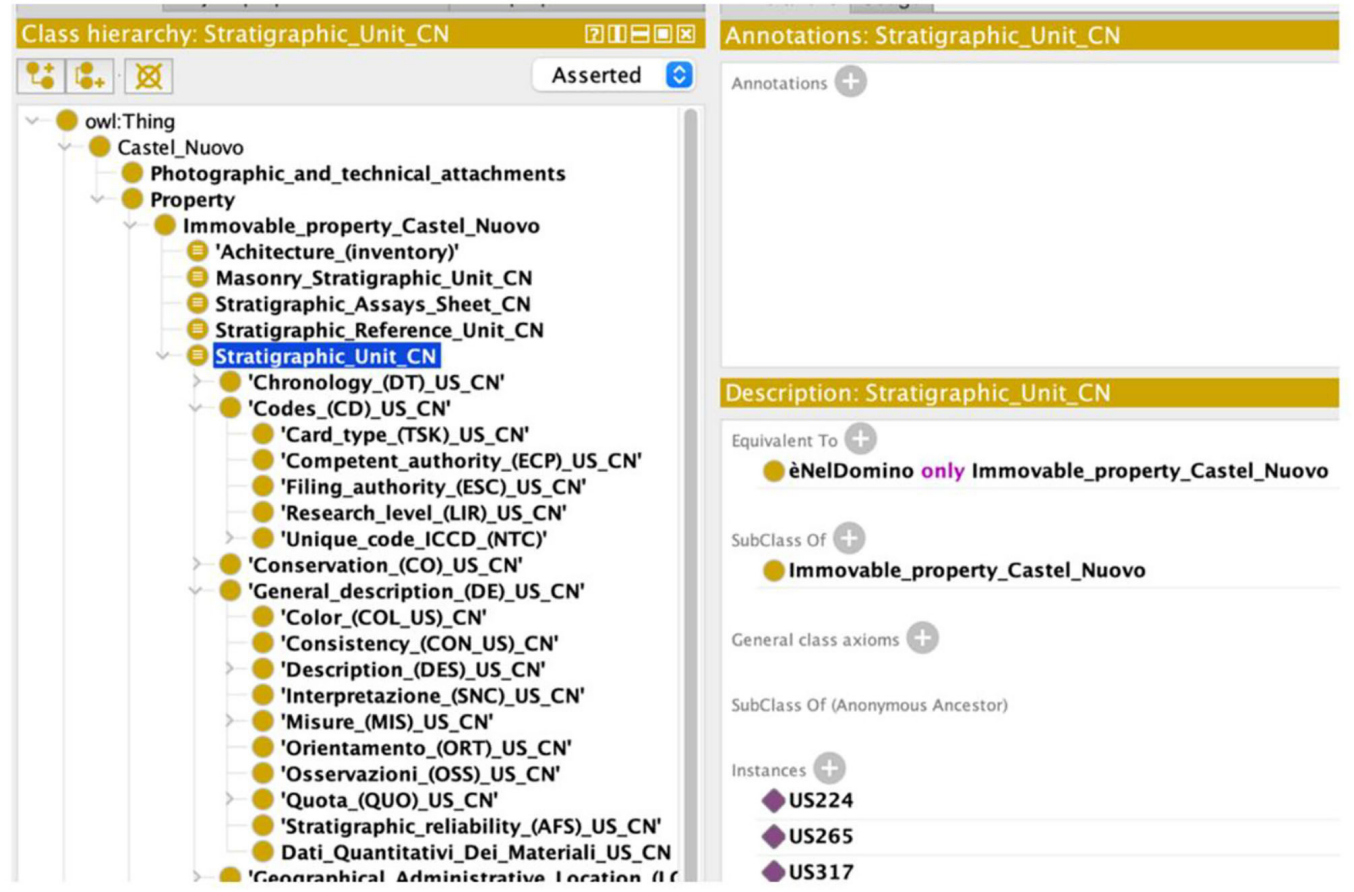
Classes of the stratigraphic unit tab modeled in the PAUN ontology.

**Figure 7:**
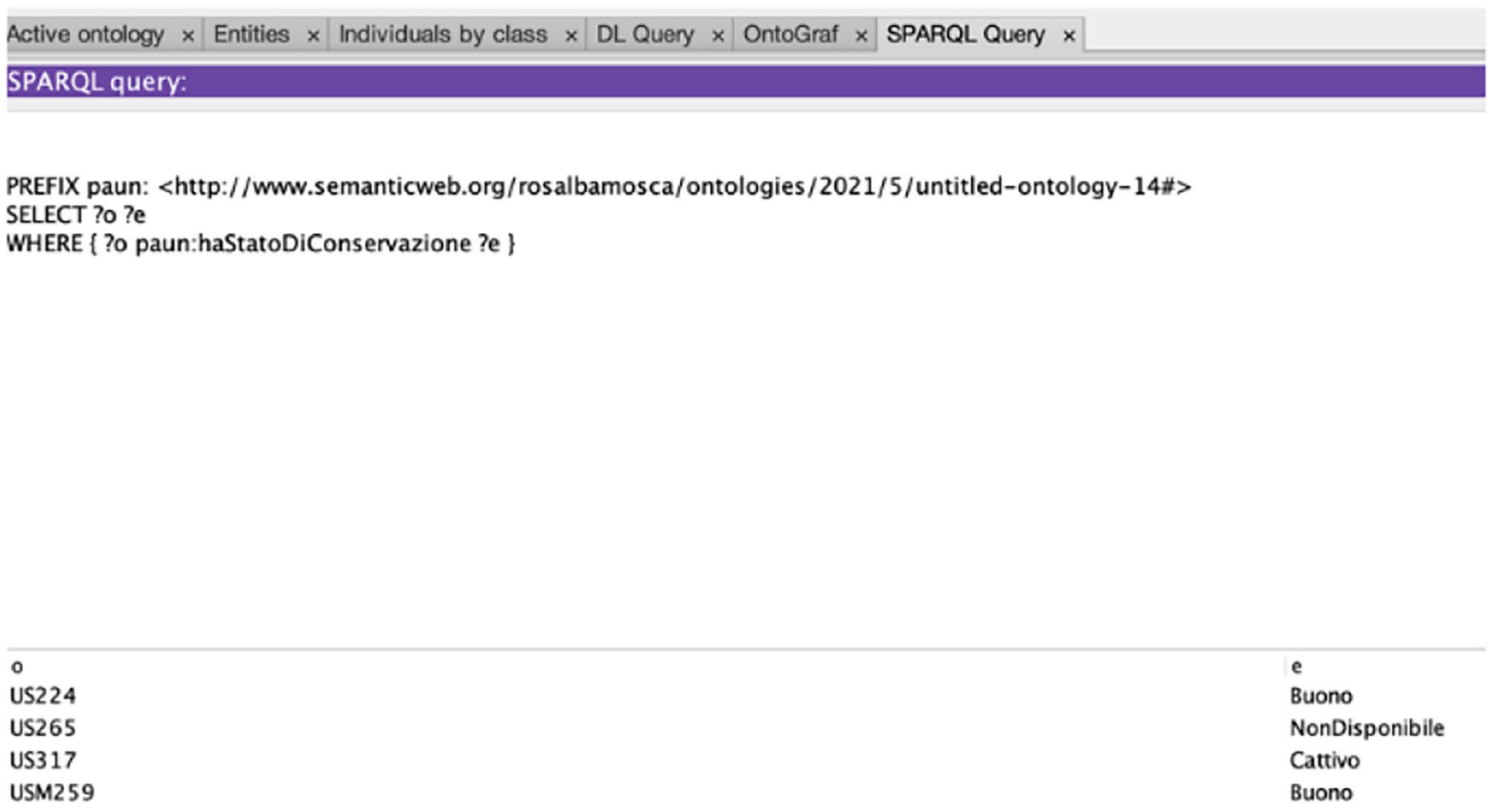
Semantic query of the PAUN Ontology on instances from the stratigraphic unit class.

Figures 6, 7 report respectively the representation of the “stratigraphic unit” in the PAUN ontology, and the query, which allows visualizing the conservation status of the stratigraphic unit.

The methodology used exploits the purpose ontology as an advantage in constructing dialogues. Correlating information, in fact, allows providing both simple information and in-depth paths based on the competence and interest of the user.

## Experimental Results

The proposed methodology's evaluation process was performed by developing a prototype based on the described architecture. The prototype consists of a server component and a front-end component. The technologies used were the Django REST framework, based on Python, to build the Rest API server-side service and the Vue.js framework for the front-end.

Even if the proposed chatbot architecture allows the system to exploit different purpose ontologies, the intent of this work is to test HeriBot behavior within the framework of the presented PAUN Purpose Ontology (PAUN_PO).

Taking PAUN_PO as a reference, our system can extract Questions/Answers pairs by systematically querying the Ontological knowledge base. In this way, for each node or group of them, the system can exploit the attributes and semantic relationships to build all possible dialogues templates, representing one or more sets of Questions/Answers pairs. These templates are organized into a dataset that contains other information such as intent and knowledge level associated with the dialogue. At this stage, the PAUN Ontology consists of about 250 nodes that present an average of 3–5 connections. Each node and each connection generates several dialog templates for a total number of 3,642. The dialog templates extracted are in Italian.

This dataset is processed through a NLP technique called tokenization, which divides a text into several parts called tokens. In this way, the text is divided into minimal analysis units (tokens), i.e., words, sentences, or parts of sentences, to enable further preprocessing steps. In our case, the text is divided into minimal sentences and, subsequently, into words. In addition, other NLP techniques such as Stemming and Lemmatization are applied. Stemming is the process of reducing a word to its root, where the root is the primary or canonical form of the original word. Lemmatization is similar to stemming; however, it uses context and lexicon-based rules to obtain the primary form of the word, called a lemma. Such processes help to standardize the input data The Question/Answer dataset is vectorized, transforming the input indices into vectors, according to the Bag of Words model, representing the Neural Network dataset (Maynard et al., 2017; Vamsi et al., 2020; Jugran et al., 2021).

A Feed-Forward Deep Neural Network (DNN) was designed (Zhu et al., 2020). The DNN was developed using the Sequential Model and composed of three fully connected layers (Chollet, 2021). In particular, the first two layers are composed respectively of 12 and eight nodes with ReLu activation function; the last dense layer is composed of 10 nodes with a softmax activation function (Manjula and Vijaya, 2020). The defined Neural Network was trained on the Question/Answer vectorized dataset, divided into Training Set (75%) and Test Set (25%). The training phase has been conducted through 150 epochs using the Adam optimizer and the loss categorical cross-entropy function, obtaining 98% accuracy and 0.0764 loss in the test phase. In this way, the system exploits the acquired knowledge to understand the questions of the users and provide the most effective answer to deliver.

**Figure 8:**
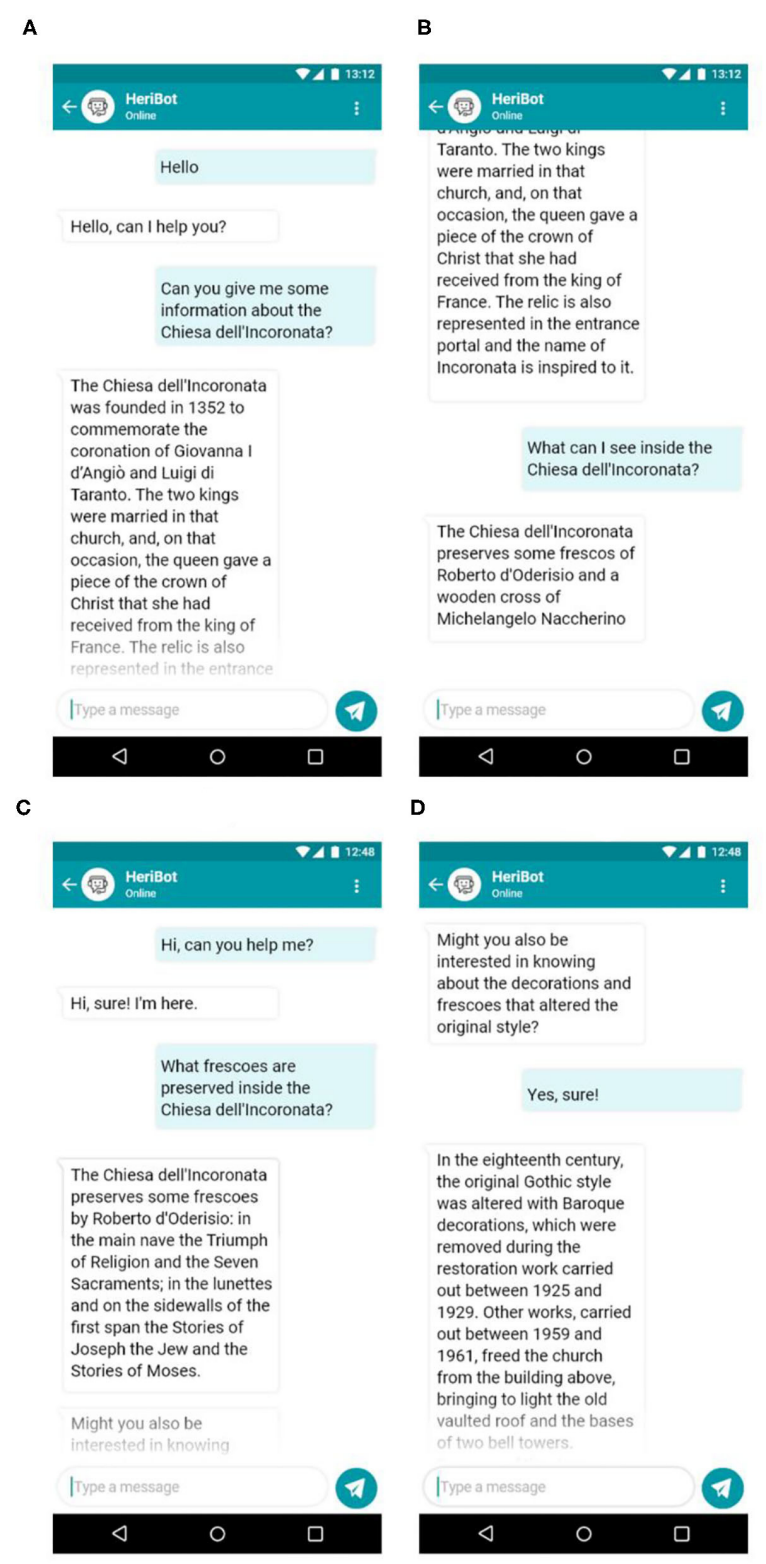
The figure reports examples of dialogues related to two categories of users. In the first example **(A,B)**, HeriBot communicates with a non-expert user, i.e., a tourist, providing general information about the Chiesa dell'Incoronata (Church of Incoronata), an asset located within the PAUN. In the second case **(C,D)**, HeriBot interacts with an expert user about the Church of Incoronata, providing more technical details.

An example of the interaction between HeriBot and users is shown in Figure 8. The figure reports examples of dialogues related to two categories of users. In the first example (8a, 8b), HeriBot communicates with a non-expert user, i.e., a tourist, providing general information about the Chiesa dell'Incoronata (a Church located within the PAUN). In the second case, HeriBot interacts with an expert user about the same Church providing more technical details (8c) and suggesting (8d) to the user to further explore the available knowledge. It is evident how HeriBot can support the expert user by providing details and information arising from associations of greater complexity.

**Figure 9:**
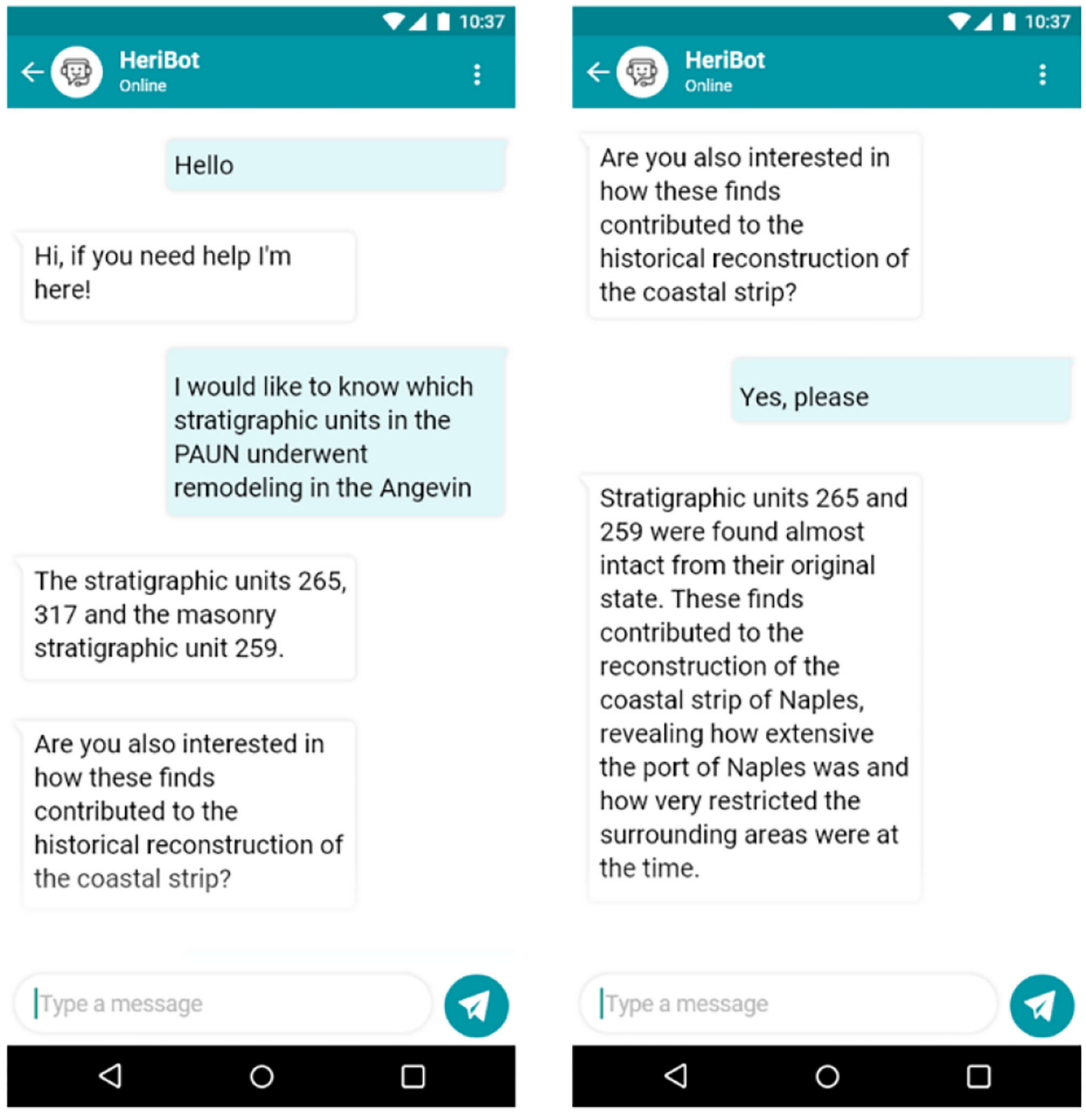
Figure shows another example of interaction with an expert user. HeriBot can provide detailed answers for the expert user, such as stratigraphic units and their conservation state.

Figure 9 shows another example of interaction with an expert user. In this case, since the user immediately requests technical and specific details, HeriBot can provide adequate and detailed answers for the target user, such as the stratigraphic units and their conservation states.

To test the Chatbot prototype to serve different categories of users, a total of about 100 users were involved, trying to divide them homogeneously by different age groups. Each participant was allowed to interact with the developed prototype, and users were divided into two main groups:

- Group 1: Standard users (85 users).

Group 1 is composed by students attending to the last (fifth) class of four different high schools aging 18/19 years old. The students were chosen as part of a program to test the results of the PAUN Project, a project funded by the European Community and the Campania Region, for the knowledge, protection and sustainable use of the cultural heritage of the Urban Archaeological Park of Naples. The experimentation was part of the students' preparation for their final exam (the so-called “maturity exam”). The students came from schools located in the Campania region but not in the city of Naples. Students have been invited to install on their smartphones, HeriBot and have been informed about the use they should make of the application during a one-day visit in the places of the park. The visits took place in four different days, one for each of the classes involved. Each visit was preceded by a presentation of the park and its places of interest held by one of the experts who collaborated in the PAUN Project. On the day following the visit, using an automatic questionnaire survey tool, the students answered anonymously the questions of the questionnaire. While answers have been anonymized, the questionnaire mandatory required answering to all questions posed.

- Group 2: Expert users (20 users).

Group 2 is made by scientists that participated to the PAUN Project. Among them there are archeologists, philosophers, art historians, and officials from the Superintendence for Architectural Heritage and Landscape of the City of Naples. All experts gave their contribution to building the original knowledge base used to design the purpose ontology of PAUN.

After the interaction experience, all users were proposed a questionnaire divided into several sections aimed at evaluating the user experience:

### Conversation

C1. The dialogues are fluid and without sudden interruptions.

C2. The system is able to understand the user's intentions.

### Reliability

R1. The system is able to understand the user's needs.

R2. The system is able to provide, in an exhaustive way, the requested information.

### Performance

P1. The interface is intuitive.

P2. The speed of response is adequate.

### Future Developments

FD1. It would be interesting to include proactivity in the developed system.

FD2. It would be interesting to use a similar approach in other scenarios.

Considering the differences between Group 1 and 2, we added to the questionnaire submitted to Group 2, a couple of questions regarding the efficient use of the knowledge base.

### Knowledge Base

KB1. The PAUN knowledge base is adequately represented in HeriChar answers

KB2. HeriBot helps to discover relations among PAUN entities.

Based on the Likert scale, each section of the questionnaire presented two statements to which five possible responses were associated: totally disagree - TD, disagree - D, Undecided - U, agree - A, totally agree - TA.

**Figure 10:**
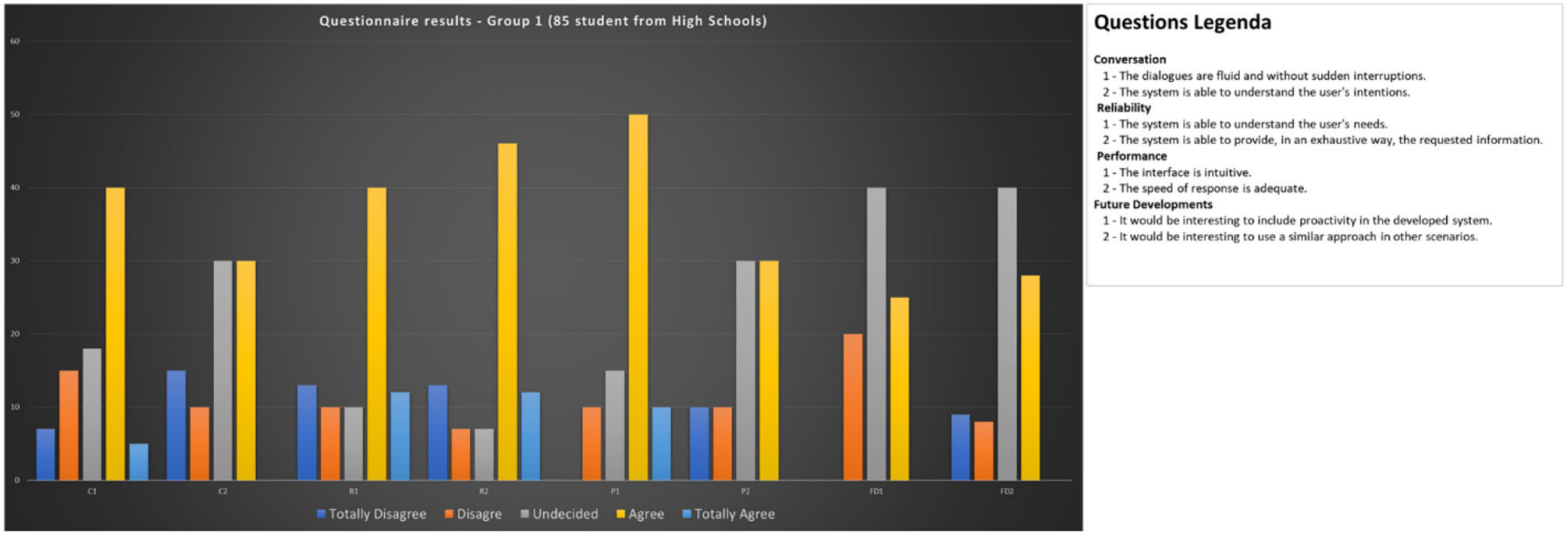
Questionnaire results for Group 1 (students).

**Figure 11:**
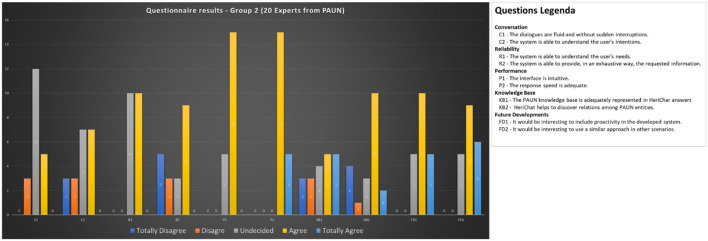
Questionnaire results for Group 2 (experts).

Figures 10, 11 report the achieved results.

## Discussion and Conclusion

Looking at the evaluations provided about statements C1 and C2, just over half of the respondents judged the fluidity of the conversation positively. This evaluation is mitigated by the perception of an adequate ability of HeriBot to provide answers able to satisfy the information needs of the user (statements R1 and R2). At the same time, this opinion becomes less positive concerning the ability to understand the real intentions contained in the user's request.

Evaluations about the interface (statements P1 and P2) are also positive for most respondents who instead judge response times as only partially satisfactory. These responses can be traced back to the fact that in Group 1 there are only young people, more accustomed to relating with typical social networking tools and demanding high performance from these tools. Finally, it is possible to highlight from the evaluations of statements FD1 and FD2 a relatively low interest in future developments. These ratings can also be clearly understood by thinking about the age of the respondents.

Comparison with the results obtained by interviewing Group 2 reinforces the considerations made thus far regarding characterization by age. Experts in Group 2 are stricter in assessing conversational characteristics (C1, C2, R1, and R2) while they judge the interface positively and are more interested in future developments.

Special attention deserves the examination of the answers to the statements related to the efficient use of the knowledge base and the ontological representation's ability to bring to light the relationships existing among the different entities present in the Park. In this case, the response of the experts turned out to be very positive.

The experimental results, although preliminary, have achieved promising results. The results showed that the system can learn and support different types of users in the fruition of the domain of interest both satisfying students and experts.

This is also encouraging from the perspective of the reusability of our approach in different scenarios.

The authors believe that the results of the experimentation conducted in the context of the PAUN Park allow us to state that the proposed approach is easily applicable in all situations in which there is a well-defined domain of knowledge from which it is possible to build an adequate purpose ontology.

Our experience has shown that it is possible to exploit the features of the purpose ontology to build dialogues more corresponding to the human user's needs.

Studying general ontologies, like ArCo, proved that such ontologies cannot be sufficiently expressive precisely because of their vastness. A large amount of data is not enough if the links between them are not expressed efficiently. Our case study experienced the importance of having a purpose ontology that expresses a specific “point of view.” It is important to remember that, in an ontology modeling aimed to train a chatbot, it is necessary to ask, “to what kind of questions the information expressed by the ontology can provide answers?” Even if an ontology does not present syntactic, logical and semantic inconsistencies between its elements, it must help provide the correct information to interested users. Equally critical is verification by domain experts. The comparison with experts in various fields (art historians, archaeologists, computer scientists, and geologists) is critical also because there is no single correct way to model a domain. An ontology, in fact, always represents a particular reality and the concepts defined in it reflect this reality.

Starting from above mentioned considerations, future developments include an investigation of methods for automatically building purpose ontologies from existing information databases concerning specific domain of knowledge. We are also planning to experiment Heribot more extensively with an incremental expansion of the PAUN Purpose Ontology. Looking at the neural network based training of the chatbot, we are planning to use a Long Short-Term memory (LSTM) approach. Unlike the feed-forward Deep Neural Networks, LSTM allows processing and performing predictions based on time series data to better elaborate long sequences of data such as conversations.

## Data Availability Statement

The datasets presented in this article are not readily available because, property of Databenc High Tech District for Cultural Heritage. Requests to access the datasets should be directed to dsantaniello@unisa.it.

## Author Contributions

All authors equally contributed to the conception and design of the study, to manuscript revision, read, and approved the submitted version.

## Conflict of Interest

The authors declare that the research was conducted in the absence of any commercial or financial relationships that could be construed as a potential conflict of interest.

## Publisher's Note

All claims expressed in this article are solely those of the authors and do not necessarily represent those of their affiliated organizations, or those of the publisher, the editors and the reviewers. Any product that may be evaluated in this article, or claim that may be made by its manufacturer, is not guaranteed or endorsed by the publisher.

## Footnotes

^1^ https://whc.unesco.org/en/list/stat#s2

^2^PAUN Project https://paun.databenc.it.

^3^ https://protege.stanford.edu

^4^ https://www.databenc.it

^5^MIBAC is the acronym for Ministero per i Beni Culturali (Italian Ministery for Cultural Heritage). ICCD stands for Istituto Centrale per il Catalogo e la Documentazione (Central Institute for Catalogue and Documentation).

## References

[B1] Adamopoulou E. Moussiades L. (2020). Chatbots: history, technology, and applications. Machine Learn. Appl. 2:6. 10.1016/j.mlwa.2020.100006

[B2] Adikari A. De Silva D. Alahakoon D. Yu X. (2019). “A cognitive model for emotion awareness in industrial chatbots,” in 2019 IEEE 17th International Conference on Industrial Informatics (INDIN), Helsinki, 183–186. 10.1109/INDIN41052.2019.8972196 27295638

[B3] Agarwal R. Wadhwa M. (2020). Review of the state-of-the-art design techniques for chatbots. SN Comput. Sci. 1:246. 10.1007/s42979-020-00255-3

[B4] Al-Zubaide H. Issa A. A. (2011). “OntBot: ontology based chatbot,” in International Symposium on Innovations in Information and Communications Technology, Amman, 7–12. 10.1109/ISIICT.2011.6149594

[B5] Arcodia C. Abreu Novais M. Cavlek N. Humpe A. (2021). Educational tourism and experiential learning: students' perceptions of field trips. Torism Rev. 76, 241–254. 10.1108/TR-05-2019-0155

[B6] Augello A. Pilato G. Vassallo G. Gaglio S. (2009).“A semantic layer on semi-structured data sources for intuitive chatbots,” in 2009 International Conference on Complex, Intelligent and Software Intensive Systems, Fukuoka, 760–765, 10.1109/CISIS.2009.165

[B7] Bakouan M. Kamagate B. H. Kone T. Oumtanaga S. Babri M. (2018). A chatbot for automatic processing of learner concerns in an online lerning platform. Int. J. Adv. Comput. Sci. Appl. 9:2018. 10.14569/IJACSA.2018.090521

[B8] Berners-Lee T. Hendler J. Lassila O. (2001). The semantic web. Sci. Am. 284, 34–43. 10.1038/scientificamerican0501-34 11396337

[B9] Carriero V. A. Gangemi A. Mancinelli M. L. Marinucci L. Nuzzolese A. G. Presutti V.. (2019). “ArCo: The italian cultural heritage knowledge graph,” in The Semantic Web - ISWC 2019 (Cham: Springer), 36–52. 10.1007/978-3-030-30796-7_3

[B10] Casillo M. Clarizia F. D'Aniello G. De Santo M. Lombardi M. Santaniello D. (2020). CHAT-Bot: a cultural heritage aware teller-bot for supporting touristic experiences. Pattern Recogn. Lett. 131, 234–243. 10.1016/j.patrec.2020.01.003

[B11] Chollet F. (2021). Deep Learning With Python. New York, NY: Simon and Schuster.

[B12] Cornevilli F. De Santo M. Dragon M. Gallo L. Troiano A. (2020). “DatabencArt and EDUBBA: digital infrastructures for cataloguing and sharing cultural heritage content,” in IOP Conference Series: Materials Science and Engineering, Volume 949, International Conference Florence Heri-tech: the Future of Heritage Science and Technologies, Bristol. 10.1088/1757-899X/949/1/012073

[B13] Dhyani M. Kumar R. (2021). An intelligent Chatbot using deep learning with Bidirectional RNN and attention model. Mater. Tod. Proc. 34, 817–824. 10.1016/j.matpr.2020.05.450 32837917 PMC7283081

[B14] Doerr M. (2009). “Ontologies for cultural heritage,” in Handbook on Ontologies, International Handbooks 463 on Information Systems, eds S. Staab and R. Studer (Berlin: Springer-Verlag), 21. 10.1007/978-3-540-92673-3_21

[B15] Ellefi M. B. Papini O. Merad D. Boi J. -M. Royer J. -P. Pasquet J.. (2018). “Cultural heritage resources profiling: ontology-based approach,” in Companion Proceedings of the The Web Conference 2018 International World Wide Web Conferences Steering Committee (Geneva: ACM), 1489–1496. 10.1145/3184558.3191598

[B16] Fryera L. K. Nakaob K. Thompson A. (2019). Chatbot learning partners: connecting learning experiences, interest and competence. Comput. Hum. Behav. 2019, 279–289. 10.1016/j.chb.2018.12.023

[B17] Gruber T. R. (1995). Toward principles for the design of ontologies used for knowledge sharing? Int. J. Hum. Comput. Stud. 43, 907–928. 10.1006/ijhc.1995.1081

[B18] Hernández F. Rodrigo L. Contreras J. Carbone F. Marcelino Botín F. (2008). “Building a cultural heritage ontology for Cantabria,” in Annual Conference of CIDOC. Athens.

[B19] Jugran S. Kumar A. Tyagi B. S. Anand V. (2021). “Extractive automatic text summarization using SpaCy in Python & NLP,” in 2021 International Conference on Advance Computing and Innovative Technologies in Engineering (Greater Noida: IEEE), 582–585. 10.1109/ICACITE51222.2021.9404712

[B20] Kakali C. Lourdi I. Stasinopoulou T. Bountouri L. Papatheodorou C. Doerr M.. (2007). “Integrating Dublin Core metadata for cultural heritage collections using ontologies,” in 2007 Proc. Int'l Conf. on Dublin Core and Metadata Applications (Seoul).

[B21] Karna M. Juliet D. S. Joy R. C. (2020). “Deep learning based Text Emotion Recognition for Chatbot applications,” in 2020 4th International Conference on Trends in Electronics and Informatics (ICOEI)(48184), Tirunelveli, 988–993. 10.1109/ICOEI48184.2020.9142879 27295638

[B22] Lokman A. Jasni M. D. (2010). One-match and all-match categories for keywords matching in chatbot. Am. J. Appl. Sci. 7:1411. 10.3844/ajassp.2010.1406.1411

[B23] Lombardi M. Pascale F. Santaniello D. (2019). “An application for Cultural Heritage using a Chatbot,” in 2019 2nd International Conference on Computer Applications & Information Security (ICCAIS), Riyadh, 1–5. 10.1109/CAIS.2019.8769525

[B24] Manjula R. Vijaya M. S. (2020). “Deep neural network for evaluating web content credibility using keras sequential model,” in Lecture Notes in Electrical Engineering, Singapore: Springer, 672. 10.1007/978-981-15-5558-9_2

[B25] Maynard D. Bontcheva K. Augenstein I. (2017). “Natural language processing for the semantic web,” in Synthesis Lectures on the Semantic Web: Theory and Technology (San Rafael, CA), 6. 10.2200/S00741ED1V01Y201611WBE015

[B26] Mendoni L. (2016). “Cultural Heritage: investing in the future,” in 1st InHeriT International Conference, May 2016, Athens. Available online at: https://www.inherit.tuc.gr (accessed March 15, 2022).

[B27] Nafis F. Yahyaouy A. Aghoutane B. (2019). “Ontologies for the classification of cultural heritage data,” in 2019 International Conference on Wireless Technologies, Embedded and Intelligent Systems (WITS), Fez, 1–7. 10.1109/WITS.2019.8723850

[B28] Noy N. F. (2004). Semantic integration: a survey of ontology-based approaches. ACM Sign. Rec. 33:4. 10.1145/1041410.1041421

[B29] Noy N. F. McGuinness D. L. (2001). Ontology Development 101: A Guide to Creating Your First Ontology (Stanford, CA: Stanford University).

[B30] Pattuelli C. M. (2011). Modeling a domain ontology for cultural heritage resources: a user-centered approach. J. Am. Soc. Inform. Sci. Technol. 2011, 314-342. 10.1002/asi.21453

[B31] Petrovski B. Aguado I. Hossmann A. Baeriswyl M. Musat C. (2018). Embedding Individual Table Columns for Resilient SQL Chatbots, Brussels: Association for Computational Linguistics. 67–73. 10.18653/v1/W18-5710

[B32] Rossana Damiano R. Lieto A. Lombardo V. (2014). “Ontology-based visualisation of cultural heritage,” in Eighth International Conference on Complex, Intelligent and Software Intensive Systems, Birmingham. 10.1109/CISIS.2014.81

[B33] Ruhanen L. (2005). Bridging the divide between theory and practice. J. Teach. Travel Tour. 5, 33–51. 10.1300/J172v05n04_03

[B34] Schmidhuber J. (2015). Deep learning in neural networks: an overview. Neural Netw. 61:2015. 10.1016/S0893-6080(14)00258-5 25462637

[B35] Shum H. He X. Di L. (2018). From Eliza to XiaoIce: challenges and opportunities with social chatbots. Front. Inform. Technol. Electr. Eng. 19, 10–26. 10.1631/FITEE.1700826

[B36] Sperlì G. (2020). “A deep learning based chatbot for cultural heritage,” in SAC'20: Proceedings of the 35th Annual ACM Symposium on Applied Computing, 935–937. 10.1145/3341105.3374129

[B37] Sperlì G. (2021). A Cultural Heritage Framework Using a Deep Learning Based Chatbot for Supporting Tourist Journey. 2021 Expert Systems With Applications. Amsterdam: Elsevier. 10.1016/j.eswa.2021.115277

[B38] Suhaili S. M. Salim N. Jambli M. N. (2021). Service chatbots: a systematic review. Expert Syst. Appl. 184:115461. 10.1016/j.eswa.2021.115461

[B39] Thomas N. T. (2016). “An e-business chatbot using AIML and LSA,” in 2016 International Conference on Advances in Computing, Communications and Informatics (ICACCI), Jaipur, 2740–2742. 10.1109/ICACCI.2016.7732476

[B40] Vamsi G. K. Rasool A. Hajela G. (2020). Chatbot: A Deep Neural Network Based Human to Machine Conversation Model (Kharagpur: IEEE). 10.1109/ICCCNT49239.2020.9225395

[B41] Wu W. Yan R. (2019). “Deep chit-chat: deep learning for chatbots,” in SIGIR'19: Proceedings of the 42nd International ACM SIGIR Conference on Research and Development in Information (Beijing), 1413–1414. 10.1145/3331184.3331388

[B42] Zhu F. Li X. Mcgoningle D. Tang H. He Z. Zhang C.. (2020). Analyze informant-based questionnaire for the early diagnosis of senile dementia using deep learning. IEEE J. Transl. Eng. Health Med. 8:2200106. 10.1109/JTEHM.2019.2959331 31966933 PMC6964964

